# Tick-Borne Encephalitis, Lombardy, Italy

**DOI:** 10.3201/eid3002.231016

**Published:** 2024-02

**Authors:** Alessandra Gaffuri, Davide Sassera, Mattia Calzolari, Lucia Gibelli, Davide Lelli, Alessandra Tebaldi, Nadia Vicari, Alessandro Bianchi, Claudio Pigoli, Monica Cerioli, Luca Zandonà, Giorgio Varisco, Irene Bertoletti, Paola Prati

**Affiliations:** Bergamo Unit, Experimental Zooprophylactic Institute of Lombardy and Emilia Romagna, Bergamo, Italy (A. Gaffuri, L. Zandonà, G. Varisco);; University of Pavia, Italy, and The I.R.C.C.S. Policlinico San Matteo Foundation, Pavia, Italy (D. Sassera);; Reggio Emilia Unit, Experimental Zooprophylactic Institute of Lombardy and Emilia Romagna, Reggio Emilia, Italy (M. Calzolari);; Milano Unit, Experimental Zooprophylactic Institute of Lombardy and Emilia Romagna, Milano, Italy (L. Gibelli, C. Pigoli);; Virology Unit, Experimental Zooprophylactic Institute of Lombardy and Emilia Romagna, Brescia, Italy (D. Lelli);; ASST Papa Giovanni XXIII Hospital, Bergamo, Italy (A. Tepaldi);; Pavia Unit, Experimental Zooprophylactic Institute of Lombardy and Emilia Romagna, Pavia, Italy (N. Vicari, P. Prati);; Sondrio Unit, Experimental Zooprophylactic Institute of Lombardy and Emilia Romagna, Sondrio, Italy (A. Bianchi, I. Bertoletti);; Epidemiology Unit, Experimental Zooprophylactic Institute of Lombardy and Emilia Romagna, Brescia (M. Cerioli).

**Keywords:** Tick-borne encephalitis, tick-borne encephalitis virus, Italy, TBE, TBEV, ticks, *Ixodes ricinus*, Europe, chamois, serological survey, clinical case, viruses, vector-borne infections

## Abstract

Tick-borne encephalitis was limited to northeast portions of Italy. We report in Lombardy, a populous region in the northwest, a chamois displaying clinical signs of tickborne encephalitis virus that had multiple virus-positive ticks attached, as well as a symptomatic man. Further, we show serologic evidence of viral circulation in the area.

Tick-borne encephalitis (TBE) is a considerable public health concern caused by the tick-borne encephalitis virus (TBEV), a member of the Flaviviridae family. This virus is classified into 5 genotypes; European, Siberian, and Far Eastern are the main types, each exhibiting distinct epidemiologic patterns and clinical manifestations (*1*). TBEV infection is mainly attributed to the bite of *Ixodes* ticks, most notably *Ixodes ricinus* in Europe (*1*). The virus primarily affects the central nervous system, leading to a range of neurologic symptoms and potential long-term complications, including death. Clinical manifestations of TBE consist of a first phase characterized by headache and fever and a second phase, where myelitis can cause altered consciousness, tremors, ataxia, and paresis (*1*).

TBEV is distributed across several regions of Europe and Asia. European TBEV is prevalent in Austria, Germany, Sweden, Switzerland, and the Russian Federation (*2*). This strain is endemic in northeastern Italy (*3*), but the rest of the country has been considered virus-free, with the exception of a single autochthonous case in Emilia-Romagna (*4*). A recent serologic screening of wild ungulates confirmed the absence of TBEV in the Piedmont region (*5*). No data on TBEV in the most populous region of Italy, Lombardy, have been published recently. Considering Lombardy’s position, bordering states (Switzerland) and regions where the pathogen is endemic, and the abundant presence of *I. ricinus* ticks (*6*,*7*), Lombardy represents an area at risk for expansion of TBEV.

## The Study

**Figure 1:**
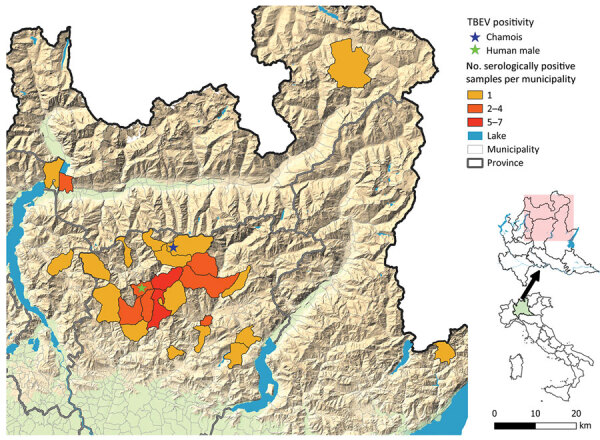
Locations of TBEV cases in a wild chamois and a human and municipalities with samples showing serologically positive results for TBEV, Lombardy region, Italy. TBEV, tick-borne encephalitis virus.

The Experimental Zooprophylactic Institute of Lombardy and Emilia Romagna (IZSLER) is responsible for the surveillance of the wild fauna in the Lombardy region. Considering the potential risk for TBEV expansion, the Institute started molecular screening of ticks retrieved from humans in Lombardy in 2019 (*8*). Since 2021, serologic analysis of wild ungulates (*9*) was added to the surveillance program, including serum samples collected in the previous year. A total of 3,555 ticks have been subjected to molecular surveillance since the start of the program (2,556 from humans, 999 from wildlife), and none were found to be positive. Out of the 1,954 examined samples from wild ungulates, 47 samples tested positive for TBEV antibodies (Table; Figure 1). Albeit not fully conclusive, those results prompted an increased alert, with attention to possible cases of neurologic symptoms compatible with TBE in animals and humans in the region.

**Table:** Results of testing for tick-borne encephalitis virus antibodies among serum samples taken from wild ungulate species, Italy*

Year	Wild ungulate species, no. positive/no. tested			
	Chamois	Roe deer	Red deer	Mouflon
2020	8/216	1/145	11/194	NA
2021	6/222	0/187	4/217	0/25
2022	4/114	3/154	7/255	0/30
2023	1/4	0/7	2/184	NA
Total	19/556	4/493	24/850	0/55

*NA, not available.

On November 28, 2022, a 49-year-old male hunter sought treatment at the Pope John XXIII Hospital in Bergamo, Lombardy, displaying clinical symptoms compatible with TBE. The man reported a tick bite in the previous month while in Val Brembana valley (Figure 1), with no recent history of travel outside of the region, but multiple mountain excursions in the study area. He reported clinical manifestations that included fever and fatigue, followed a few days later by neurologic symptoms (lack of coordination and equilibrium), which supported a 2-phase clinical picture compatible with TBE (*1*). Serologic exam for TBEV resulted in positive readings for both IgG and IgM. The patient’s clinical picture improved spontaneously, and he was discharged on December 12, 2022. Since that time, he has reported myalgia, fatigue, impairment of concentration, and memory lapses.

**Figure 2:**
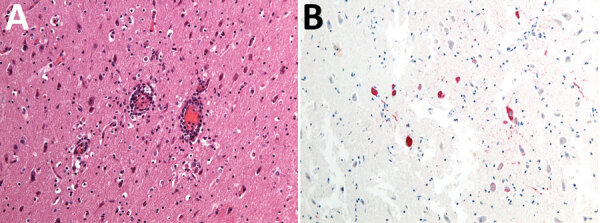
Histopathologic findings from the brain of a wild chamois with tick-borne encephalitis virus found in the Lombardy region of Italy in May 2023. A) Severe, chronic, nonpurulent meningoencephalitis characterized by perivascular lymphohistiocytic cuffs. Hematoxylin-eosin stain; original magnification ×20. B) Neuronal positivity for tick-borne encephalitis virus. Immunohistochemistry; original magnification ×20.

On May 12, 2023, in Carona, Bergamo Province, Lombardy region (Figure 1), a hunter encountered a chamois (European goat-antelope) that had neurologic symptoms of ataxia, muscle tremors, incoordination, and frequent swallowing. The chamois was killed and conferred to the IZSLER, where we performed necropsy and collected blood and organs for further examination. Postmortem examination revealed poor general condition, absence of adipose tissue, and incomplete molt. Necropsy did not reveal mechanical trauma or ingestion of poisonous food. A massive tick infestation was presen; some ticks were clustered, but others were scattered on various areas of the body. At the lung level, pleuro-costal adhesions and parasitic nodular lesions were evident. Other observations included gastrointestinal nematodes, pallor of the kidney parenchyma and cribrous appearance of the cortical surface, hypertrophy of the adrenals, and pallor of the liver, including focal irregular, whitish lesions on the surface. Histopathology revealed severe, chronic, nonpurulent meningoencephalitis, characterized by perivascular lymphohistiocytic cuffs, neuronal necrosis, and satellitosis (Figure 2, panel A). Immunohistochemical analysis showed neuronal positivity for TBEV (Figure 2, panel B). An attempt to culture the virus from brain tissue on Vero E6 cells (IZSLER Biobank code BSCL87, http://www.ibvr.org) was unsuccessful.

**Figure 3:**
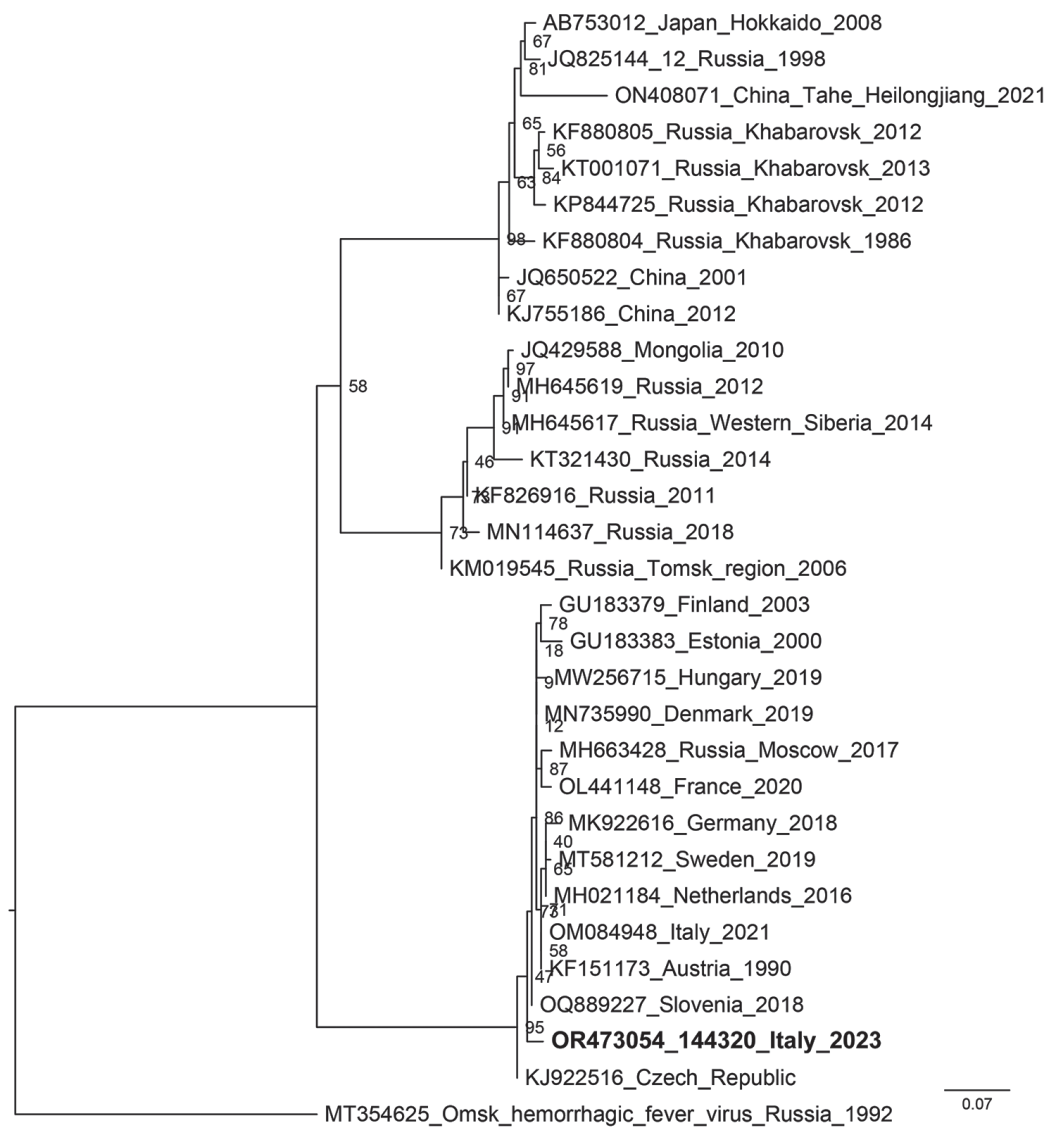
Phylogenetic tree of a representative tick-borne encephalitis virus (boldface) from samples collected from a wild chamois and ticks in the Lombardy region of Italy. Tree shows the relationship between the obtained sequence of a 224-bp portion of the nonstructural 5 gene and reference sequences from GenBank (accession numbers, country, and year of isolation provided). The phylogenetic analysis was performed on the homologous sequences by the maximum-likelihood method using IQ-TREE software (http://www.iqtree.org), after alignment.

The animal’s blood was used for molecular and serologic investigations, which both showed evident TBEV positivity, as did a pool of organs and brain. The threshold cycle of the PCR of the organ pool was low, suggesting a high viraemia. A total of 26 ticks were found attached to the body, all identified morphologically as adult *I. ricinus* ticks, 12 of them male and 14 partially engorged female. All retrieved ticks were subjected to TBEV PCR; 4 male and 8 female ticks showed clear positivity. We Sanger sequenced PCR products (224 nt) from the chamois and from the ticks; sequences were all identical (Genbank accession nos. OR473050–4) so we used 1 representative for phylogenetic reconstruction (Figure 3). The phylogenetic tree shows that the novel sequence falls within sequences representing the European genotype (*10*). 

In parallel, we performed bacteriologic examination of the viscera of the chamois and tested for *Anaplasma phagocytophilum*, *Babesia* spp., and pestiviruses. All test results were negative. We also screened the ticks for other pathogens, namely *Borrelia* spp., *Babesia* spp., *Rickettsia* spp., *Francisella* spp*., Coxiella burnetii*, and *Anaplasma*, as previously performed (*11*,*12*). All results were negative, except for 2 females found positive for *Rickettsia helvetica* and 1 found positive for *Borrelia miyamotoi,* all confirmed by Sanger sequencing.

## Conclusions

We report TBE cases in a human and in a chamois in the Lombardy region of Italy, as well as molecular positivity in *I. ricinus* ticks. A clinical case in a chamois is especially noteworthy given recently reported clinical cases of TBEV in other wild and domestic animals, including ruminants (*13*), and the steady increase of wild ruminants in the Alps (*14*). The high viraemia of the chamois we studied, together with the TBEV positivity of multiple attached ticks, suggests a potential role in the maintenance of the virus, one generally not considered (*13*,*15*). Our finding of TBE-positive *I. ricinus* male ticks with sequences identical to female ticks is compatible with transstadial and venereal transmission; female tick positivity could also be explained by cofeeding. When considered alongside the high amount of virus detected in the chamois, the findings from our analysis of the attached ticks corroborate the existence of a complete cycle of infection and a potential role of this animal as reservoir.

Results from our investigation indicate the presence of TBE in northwestern Italy and suggest the need for increased awareness of the westward spread of TBEV, now present in most of the central Alps. The severity of the reported human case highlights the importance of raising the awareness of stakeholders and high-risk portions of the population (e.g., hunters, excursionists, and farmers) to promote vaccination and control of raw milk and traditional cheesemaking. Taking immediate preventive measures in the most at-risk areas will help prevent subsequent clinical cases of TBEV in humans.
